# Factors influencing the guest-host knowledge transfer for pro-poor tourism in China’s western ethnic-minority areas

**DOI:** 10.1371/journal.pone.0322370

**Published:** 2025-04-29

**Authors:** Jin Wu, Shiyi Tang, Rui Zhang

**Affiliations:** College of Tourism & Landscape Architecture, Guilin University of Technology, Guilin, Guangxi, China; Zhejiang Shuren University, CHINA

## Abstract

The crucial transfer of knowledge through pro-poor tourism enables poor people to enhance their own endogenous-development dynamics. However, it is difficult to use existing knowledge transfer theories to explain the behavior of ethnic-minority villagers in western China, due to low levels of education and smallholder household-based tourism operations. The present study uses MOA theory to examine the influence of motivation, opportunity, ability, and trust on the guest–host transfer of knowledge in ethnic-minority villages in western China. Here, knowledge transfer opportunities are shown to be the main driver of the MOA framework, positively affecting the knowledge transfer effect. Knowledge transfer motivation and knowledge absorptive capacity have an indirect, positive impact on knowledge transfer opportunities. Although local people have a high level of trust in external knowledge sources, trust does not have significant moderating effect between knowledge transfer opportunities and effects. These findings can be used to strengthen the transfer of valuable knowledge to villagers and to cultivate rural tourism talents.

## Introduction

In the late 1990s, the U.K. Department for International Development (DFID) proposed the concept of pro-poor tourism (PPT) and suggested that PPT strategies could generate net beneﬁts for poor people [1]. An increasing number of developing countries are incorporating rural tourism into their national economic agendas as a poverty alleviation tool to improve the wellbeing of local people [2]. According to Jiang et al. (2022), small-scale non-agricultural activities represent an important source of revenue and employment for poor people in rural areas, creating new local venues for economic growth [3]. Tourism is a key activity in economically disadvantaged areas with rich tourism resources [4,5] and rural tourism makes an important contribution to rural economic growth [6]. However, some critics argue that PPT does not always benefit the poor people as intended. Yu et al. (2019) suggested that PPT projects may exacerbate social inequalities, as resources are often controlled by local elites rather than benefiting poor populations [7]. Pham and Nugroho (2022) found that PPT in Indonesia may lack resilience to external shocks, hindering its continuous support for poor people during the COVID-19 pandemic [8]. Conversely, proponents argue that PPT can facilitate development in impoverished regions through policy design and community participation [9]. Pang et al. (2024) found that community-led PPT projects significantly increased income for poor participants [10]. Ultimately, PPT’s effectiveness hinges on policy support, community involvement, and equitable benefit distribution. China’s western villages abound in beautiful landscapes and natural resources, while offering the change to experience unique ethnic-minority cultures. For many decades, local people have worked in agriculture and animal husbandry. Their villages have become impoverished, lacking the development opportunities introduced through industrial agglomeration and infrastructure construction in central and eastern China. The development of rural tourism in western Chinese therefore represents a significant opportunity to promote economic growth, alleviate local poverty, and improve ecological environments. However, the picture is far from complete. Many parts of western China are failing to promote tourism effectively. Although rural tourism has the potential to become be a major force in rural economic development, that potential has not yet been fully realized [11].

The equitable distribution of tourism revenues to local communities, facilitated by PPT, can significantly enhance a tourism destination’s sustainable development capacity, thereby promoting local economic prosperity and social stability [10]. However, little attention has been paid to human resource management in rural areas [12]. In recent years, the balance between knowledge and resources has shifted—knowledge is now the key factor that determines the standard of living, outpacing land, tools, and labor [13]. According to Rao et al. (2024), the main obstacle impeding PPT remains the grave lack of knowledge and skills in poor populations [14]. A lack of tourism knowledge and skills prevents rural people from participating actively in tourism planning and development [15]. As efforts to strengthen people’s abilities play an important role in the implementation of pro-poor strategies [16], capacity building has been identified as a critical factor in knowledge-driven PPT interventions. There is now a focus on the transfer of knowledge via PPT as a means of strengthening the self-development capacity of very poor populations. When villagers change their identities, evolving from traditional agricultural workers into tourism service providers, they must acquire tourism service knowledge. Knowledge transfers increase their ability to manage tourism businesses, revitalize the local talent pool, and enable the development of rural tourism [17]. Knowledge transfers also improve endogenous development dynamics. As a result, efforts to develop the economy through the transfer of knowledge to poor rural laborers are gaining more traction in governance and research.

With the advent of the knowledge economy era, knowledge transfer has become a hot research topic in the field of tourism. Scholars have extensively discussed the knowledge transfer both within and between organizations, and knowledge transfer in rural tourism. When considering knowledge transfer within tourism firms, Tsai (2021) found a positive correlation between individual-level knowledge absorptive capacity and psychological ownership [18]. Additionally, Türkmendağ and Tuna (2021) highlighted that empowering leadership significantly influences the creation, sharing, and application of knowledge among followers within the hotel management system [19]. In studies focused on knowledge transfer between organizations, Weidenfeld et al. (2010) demonstrated that spatial proximity, product similarity, and market similarity generally enhance knowledge transfers and innovation spillovers among attractions at local and regional scales [20]. Furthermore, Chen and Lee (2017) identified four distinct categories of market knowledge within the travel industry, encompassing customer knowledge, employee knowledge, competitor knowledge, and partner knowledge [21]. Larkin (2020) emphasized the occurrence of knowledge creation and assimilation within clusters in hospitality multi-national enterprise [22]; Raisi et al. (2020) conducted a network analysis to investigate inter-organizational knowledge transfer in Western Australian tourism [23]. They discovered that the knowledge network of the tourism sector exhibits strikingly similar topological properties to the hyperlink network of the destination. Scholars who study the transfer knowledge to villagers engaged in rural tourism have identified the types of ability acquired from knowledge transfer, such as tourism service and management ability, technology application ability, and market judgement ability [17], and explored that knowledge transfer promotes villagers’ positive attitudes toward rural tourism [24] and improve business effectiveness [25]. Wiltshier et al. (2014) have explored the transfer of knowledge from universities to rural tourism areas and found that villagers are exposed to marketing and finance expertise [26]. According to Longart et al. (2017), knowledge is transferred from universities to villagers in three stages: initiation, execution, and “the leap” [27]. This process involves multiple stakeholders, including higher-education institutions, tourism managers, teachers, students, and people in rural communities. Rao et al. (2018) have analyzed the transfer of knowledge between external workers arriving in a village and villagers engaged in rural tourism, drawing conclusions about the technical process of knowledge transfer and the reproduction of social relationships [28]. Zhou (2019) has analyzed knowledge transfer mechanisms in rural communities and found that social capital, comprising interpersonal trust, reciprocal cooperation, and shared vision have an important impact on knowledge transfer [29]. Zhang and Ji (2022) [30] and Zhang et al. (2023) [2] have advanced the notion that the initial cohort of rural tourism entrepreneurs assumes a pivotal, leading, and exemplary role during the developmental phase of knowledge transfer. It has been elucidated that technological progress acts as the primary driving force behind the knowledge transfer system in rural tourism. Current research endeavors have successfully integrated the dimension of knowledge transfer into the broader research framework of innovations within the tourism sector. Furthermore, researchers have systematically examined the various factors that influencing the knowledge transfer process and its distinctive role in bolstering the performance of organizations operating within the tourism industry. However, many knowledge transfer studies have focused on medium-sized and large tourism businesses at the organizational level, paying insufficient attention to the transfer of knowledge to rural people involved in rural tourism. Scholars have analyzed the content, process, and factors involved in the transfer of knowledge from external entering workers and universities to villagers working in rural tourism industries. The implementation of PPT is committed to strengthening the self-development capacity of poor populations, in which knowledge transfer has an important role. Few studies have explored the factors that influence knowledge transfer from the perspective of villagers engaged in PPT.

It is generally argued that the best way to upgrade the capacity of a poor population is by investing in formal education, including technical extension and vocational education [31]. However, this lopsided dependence on educational investment may result in transpositions of recipients and content, as most people involved in tourism development are beyond schooling age. Explicit knowledge is easily documented and communicated [32], while tacit knowledge is difficult to express [33]. Tourism professionals need implicit knowledge and skills that cannot be acquired through classroom instruction. For this reason, the transfer of knowledge in work situations is an effective way for villagers to acquire knowledge.

As rural tourism develops, college students, journalists, tourists, external tourism staff, and other stakeholders enter the area and communicate with local people. Opportunities for the transfer of knowledge to villagers emerge during contact between stakeholders and residents, including formal training and “learning by doing” [34,35]. Stakeholders, as guests and external sources of knowledge, transfer tourism service knowledge, management skills, and innovative ideas to local people through interactions [36]. This guest–host knowledge transfer effectively addresses issues such as the lack of tourism service knowledge and skills, limited learning channels, and insufficient application of new knowledge [17]. Knowledge transfers help local micro- and small-scale rural tourism businesses succeed and promotes tourism destination development [37,38]. In the process of knowledge transfer in tourism destinations, learning is not only an effective way for employees to acquire knowledge, but also an important channel for tourism enterprises to transfer knowledge [17]. Local people learn from external sources to understand the needs of tourists, the nature of local tourism resources, the tourism reception infrastructure, service and reception skills, personal working conditions and abilities, the role of government and corporate experts, organizational management and operation models, input and output benefit distribution models, resource and environmental protection, and innovation development. Additionally, Valeri and Baggio (2020) argue that labor mobility plays a key role in knowledge transfer within tourism destinations [39]. As tourism practitioners frequently change jobs, the knowledge gained from previous roles is also transferred. These practitioners then acquire “new” tacit knowledge applicable to new positions, a process requiring time and relying heavily on learning-by-doing experience [40]. This knowledge is invaluable for tourism enterprises.

When researching the transfer of tacit knowledge, based on work context, we cannot apply current knowledge transfer theories directly, without considering the perspective of rural people. The ethnic-minority villages in western China are mostly located in mountainous areas, plateaus, pastoral zones, and forest regions, where the rural industries are primarily based on agriculture and animal husbandry. These villages are characterized by ethnic diversity, a relatively closed natural economic environment, poverty, weak human capital, and incomplete infrastructure. Therefore, the limitations in culture, education, and infrastructure pose unique challenges to knowledge transfer for villagers. First, disparities in perceptions of knowledge value and sharing across ethnic groups present challenges for adopting novel external ideas, technologies, and knowledge within certain ethnic minority village communities [38,41]. Second, the China Rural Statistical Yearbook 2022 [42] indicates that average education for rural residents in China is 7–9 years. Ethnic-minority villagers generally have lower educational levels and limited capacity to acquire and apply new knowledge; prior to tourism development, many could not even speak Mandarin. Moreover, remote geographical locations hinder infrastructure development in ethnic-minority villages in western China. Inadequate infrastructure, such as water, electricity, roads, and communication networks, limits access to external knowledge sources, as well as knowledge acquisition and dissemination. Currently, research lacks in-depth analysis of the key factors influencing tourism knowledge transfer activities in ethnic-minority rural settings from the viewpoint of local villagers. To fill this research gap, the study uses the MOA model to analyze and test empirically the effects of motivation, opportunity, ability, and trust on PPT-based guest–host knowledge transfers in ethnic-minority regions in western China. Our findings have significant value because they enrich knowledge transfer related theories, enhancing the transfer of tourism knowledge to rural people and achieving the purpose of PPT. Furthermore, the ethnic-minority villages in western China are known for their beautiful natural landscapes and unique ethnic cultures, but most are located in remote areas and are economically underdeveloped. The present study’s conclusions hold potential for informing tourism knowledge transfer strategies in rural areas sharing analogous characteristics, particularly in ethnic-minority and indigenous village communities located in Southeast Asia (e.g., India, Vietnam, Thailand, Laos, and Cambodia), as well as parts of Africa and Latin America. These villages share similarities with the ethnic-minority villages in western China in terms of natural beauty, traditional culture preservation, and rural tourism development, but they also face challenges such as underdevelopment and weak infrastructure. Specifically, this study emphasizes the importance of villagers developing professional skills through acquiring new tourism knowledge; the crucial role of government and non-profit organizations in augmenting villagers’ learning opportunities, willingness to participate in rural tourism, and participation abilities; and the necessity of improving rural infrastructure to promote villagers’ career transitions, providing constructive strategies and methods for other underdeveloped or culturally unique regions.

## Theory and hypotheses

### MOA framework

The basis for this research is the well-known motivation/opportunity/ability (MOA) framework, which has been applied in various management disciplines. The MOA framework is well established as a theoretical foundation for work performance investigations [43,44]. The origins of the MOA framework lie in the theoretical discourse among industrial psychologists, who have traditionally viewed performance as a function of training and selection, which sharpen an individual’s ability to perform [45]. From an economic perspective, personal behaviors are primarily motivated by self-interest. As people tend to work hard to maximize individual utility [46,47], social psychologists initially emphasized the motivational component of performance [48]. Subsequently, an additional opportunity was added to the framework to capture all of the exogenous factors that prevent individuals from performing well [43]. Such factors have often been described as situational or operational constraints [45,49]. In recent years, the MOA framework has been applied to knowledge-management practices [50,51]. “Broadly speaking, motivation captures the individual’s willingness to act; opportunity represents the environmental or contextual mechanisms that enable action; ability represents the individual’s skills or knowledge base related to the action” [52]. The three MOA factors that influence knowledge transfer related behavior have been widely acknowledged; to obtain maximum benefit, researchers aiming to design and implement a knowledge management system must consider these factors [50].

The MOA framework highlights the importance of motivation as a driver of action [53]. Here, the motivation is the local villagers’ desire to acquire new tourism related knowledge and skills. The Chinese west has long been a fortress for ethnic minorities and foreign nationals engaged in migration and exchange. Many nationalities have settled here, creating a point of convergence for eastern and western civilizations and ethnic cultures. In terms of population distribution, this area is characterized as heterogeneous, due to the number of ethnicities, but with a homogeneous indigenous local population. As they are interdependent, often merging in their coexistence, these groups have developed unique and variegated ethnic production lifestyles, in keeping with highly enclosed environments, nomadic grassland economies, and small-scale self-sufficient peasant economies [54].

Plurality and multi-layered ethnic social environments have enabled researchers to differentiate between ethnic groups in different parts of the Chinese west, based on their values, spirit of survival and competitiveness, adaptability to modern society, and attitudes toward technological transformation. Since ethnic cultures have penetrated all aspects of this society, sustaining traditional customs, lifestyle habits, and farming livelihood patterns [55], it can be very hard for such exclusive forms of culture, developed in isolation, to accommodate, communicate with, and accept more advanced technology and livelihood patterns. Some groups have even boycotted their introduction [56].

Since 2006, China has gradually built roads into the countryside. New modes of information transmission, such as radio and television, have been spreading to the villages for more than 20 years. For Chinese minority villages, which have long been isolated from the rest of the world, these links to the outside world have not fundamentally changed the poverty and wealth of local villagers, who seem overlooked by the ever changing world outside [57]. Due to remote geographical locations and economic underdevelopment, the carriers of rural knowledge transfer, such as rural libraries, rural schools, the internet, and the system of science and technology commissioners, are difficult to establish in ethnic-minority villages [58]. Consequently, these carriers are unable to effectively support rural tourism development within these communities.

Low levels of community participation impede poverty reduction through tourism [59]. Kunasekaran (2022) has validated the significance of residents’ perceptions of the impact of community-participation opportunities [60]. The perceived economic effects of resident-initiated tourism provide strong motivation and promote participation [61]. There is a positive relationship between knowledge transfer motivation and reward [62]. According to Aalbers et al. (2013), an intrinsically motivated individual acts out of personal choice and interest [63]. In China, the central government plays a steering role in guiding rural tourism in the desired direction, while local government plays an enabling role by managing tourism practices directly and coordinating with businesses and residents to provide services and solve problems [64]. Some non-profit pro-poor organizations are committed to helping people in extremely poor rural areas [65,66]. In the 1990s, when rural tourism emerged in China, some photographers and explorers trekked to western ethnic-minority villages on foot. They became the first tourists in the villages. Later, a few rural people began to engage in tourism hospitality and earned a good income. The promotion of and support for rural tourism by governmental and pro-poor organizations have made some villagers realize that rural tourism is a way out of poverty. They have also been inspired by seeing other villagers provide paid services to tourists. These developments have stimulated a profound desire for a change in livelihood patterns among local ethnic-minority villagers. Thus, strong economic motivation and the desire to change careers are prompting villagers to search for external knowledge and recognize knowledge transfer opportunities. We therefore propose the following hypothesis:

**H1:** Knowledge transfer motivation has a significant positive effect on the recognition of knowledge transfer opportunities.

Opportunity is the absence of environmental barriers to the transfer of knowledge. Work performance theory suggests that motivation, opportunity, and ability play complementary roles in influencing behavior [67]. Without opportunity, motivation and ability cannot lead to knowledge sharing behavior. Knowledge transfer opportunities encompass free training projects provided by tourism enterprises, government organizations, and non-profit pro-poor organizations, as well as informal interactions between tourism enterprise employees, experts, PPT volunteers, tourists, other stakeholders, and local villagers involved in rural tourism [68]. During the process of guest–host knowledge transfer, the guest is the PPT related group that owns the knowledge, while the recipients are local ethnic-minority villagers involved in the tourism industry. Rural tourism is expected to create commercial opportunities that profit the local community [69]. Communications between the guest and host focus on ways to increase the income of local minority villagers who engage in rural tourism services; this initiates a two-way tourism transfer interaction in which knowledge is delivered by external sources to local minority villagers. Thus, we propose the following hypothesis:

**H2:** Knowledge transfer opportunities have a significant positive impact on the knowledge transfer effect of tourism.

In this paper, the fieldwork results reveal that, in remote, closed, chronically poor, traditional, and knowledge starved ethnic-minority villages, the original and tourism knowledge frameworks are two isolated systems. For villagers, the shift in identity and occupation is a huge challenge, involving knowledge, experience, ability, morality, ethics, and spirituality. The knowledge skills of previous generations were based on the land, forest, animal husbandry, manual labor, and farming. It is therefore difficult for villagers to successfully embed a system of advanced tourism imported from outside into their existing knowledge framework and to gain full recognition and acceptance of the associated concepts, emotions, values, ethics, and spirituality. In general, the tourist’s need for food, accommodation, transportation, tourism, shopping, and entertainment represent the most direct fit with local clothing, food, accommodation, transportation, and hospitality traditions passed down from generation to generation by ethnic-minority villagers. The main difference is that the original knowledge reflects the obligatory and reciprocal “ethics of hospitality”, while tourism services introduce the new commercial and profit oriented “ethics of hospitality”. A hospitable atmosphere and homemade cuisine have become the most attractive rural-tourism products offered in rural villages. Villagers serve tourists as their guests and try to create a home atmosphere, promoting customer satisfaction [70].

People’s evolving life experiences continuously affect the development and shape of their knowledge and skills [71], which the essential drivers of behavior and preferences [72]. The villagers’ knowledge absorptive capacity allows them to recognize and integrate advanced external tourism knowledge into their own theoretical framework and apply it to practical work. This difficult to transfer tacit knowledge requires villagers to have a certain amount of prior knowledge, professional perception, and knowledge application. We therefore propose the following hypothesis:

**H3:** Knowledge absorptive capacity has a significant positive effect on knowledge transfer opportunities.

### The moderation effect of trust

Zand (1972) has described trust-building as actions taken by individuals that increase vulnerability to the behaviors of others outside their control; such actions can cause individuals to suffer if that vulnerability is abused [73]. Although studies of the relationship between trust and knowledge transfer have occurred under different circumstances, most conclude that when levels of trust among knowledge transfer participants are relatively high, one side is willing to take higher risks, dropping its defenses and believing that the other party will reciprocate in kind [74]. Trust reduces costs and promotes knowledge transfer [75].

Fundamentally, the villagers’ determination to participate in tourism services is an attempt to change their occupations and identity, shifting from a primary industry based on agriculture to a tertiary industry based on tourism. Individually, their identities change from villagers working the land to service providers and tourism hosts. This conversion involves the investment of time, energy, materials, and money. There are also huge investment risks and opportunity, learning, management, and maintenance costs. Trust is a key element and the basis for cooperation between villagers and stakeholders [76]. It is crucial to the cooperation and networking processes that characterize integrated rural tourism [77]. Trust building in a network occurs through information and knowledge sharing and commitment [78]. In western China, the essence of knowledge transfer is the process by which ethnic-minority villagers learn new occupational skills and complete a change of occupation. There are many uncertainties involved in giving up original vested interests and prospective incomes to become tourism service providers, potentially earning more than their original status; these impact the decision to change occupation and status. Trust in government positively affects the villagers’ intention to participate in rural tourism via perceived usefulness [79], good personal relationships, and mutual trust [80]. Trust significantly moderates the positive effects of effective training on knowledge transfer outcomes [81] and of attitudes towards knowledge transfer on knowledge transfer behavior [82]. In ethnic-minority villages, trust acts as a contextual factor, facilitating villagers’ active utilization of knowledge transfer opportunities, which in turn leads to knowledge transfer behaviors. If trust is considered as a direct antecedent, it implies that it will directly drive knowledge transfer behaviors, which is inconsistent with knowledge transfer practices; if trust is considered as a mediator, it will explain how knowledge transfer opportunities, ability, or motivation affect the knowledge transfer effect through trust, which does not align with the core logic of the MOA framework. In summary, the present study treats trust as a moderating variable in the effectiveness of knowledge transfer and opportunities for knowledge transfer. We therefore propose the following hypothesis:

**H4:** Trust significantly and positively moderates the relationship between knowledge transfer opportunities and knowledge transfer effects.

### Research framework

**Fig 1:**
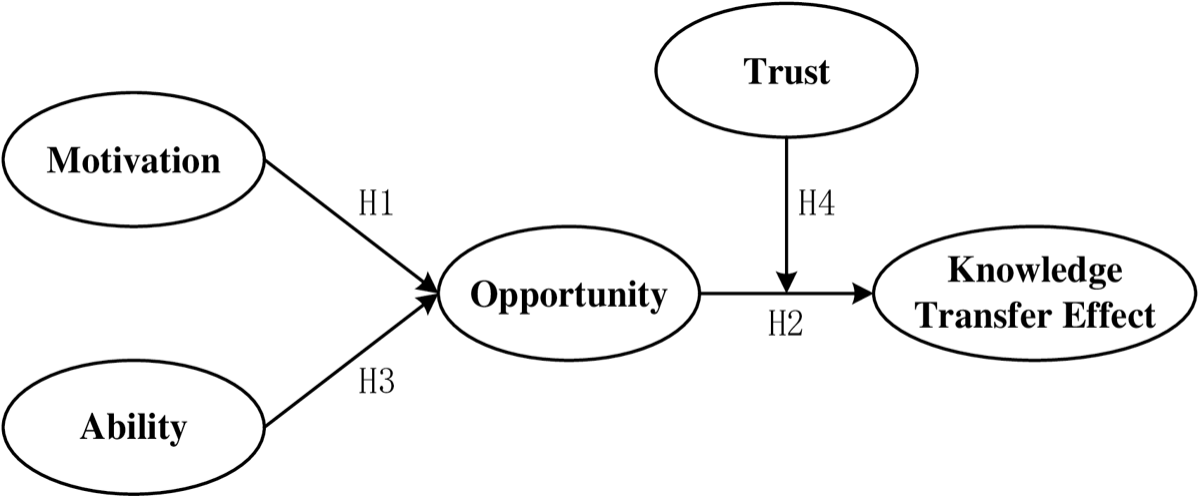
The research framework: factors that influence guest–host knowledge transfer via PPT.

Based on the discussion above, Fig 1 presents the model tested in the present study. The next section will analyze the factors that influence tourism knowledge transfer and differences in impact.

## Methods

### Sample and procedure

Western China has 44 ethnic minorities, comprising 8.89% of the total population of China. Of these, Zhuang people account for 12.54% of the minority population [83]. Together, the Zhuang, Dong, Miao, and Yao ethnic minorities account for a relatively high proportion of the minority population in western China. To ensure data reliability, researchers conducted more than ten visits to 11 villages of the Yao, Zhuang, Dong, and Miao ethnic groups (areas where rural tourism is well-developed) between 01/05/2020 and 18/06/2021. The specific survey locations were Yunlong Nuodeng Ancient Village, Weixin Wanzi Miao Village, Yiliang Yunchong Miao Village, Longji Terraces Scenic Area, Jinxiuzhongzhan Yao Village, Xing’an Longtang Village, Chengyang Eight Villages, Liping Zhaoxing Dong Village, Leishan Langde Miao Village, Xijiang Qianhu Miao Village, and Libo Yaoshan Ancient Village. These villages, inhabited by ethnic minorities, are located in remote areas and were once economically underdeveloped, but possess beautiful natural scenery and rich, diverse ethnic-minority cultural resources, making them suitable for developing rural tourism. The surveyed areas reflect the general characteristics of ethnic-minority villages in western China. In practice, after years of rural tourism development, the above 11 villages have become mature tourism destinations, exploring successful models of rural tourism development in these areas. Most villagers have participated in rural tourism hospitality and acquired benefits from PPT. The focus of this study is on these groups, and the success of these villagers provides valuable insights for knowledge transfer in other ethnic-minority villages.

The survey was conducted using a questionnaire and the research subjects were rural tourism practitioners in ethnic villages. Each questionnaire took more than 30 minutes to complete and all data were collected on-site. This study was approved by the Ethics Committee of the College of Tourism & Landscape Architecture, Guilin University of Technology, under approval number 2020006. The participants in this study were all adults, and written informed consent was obtained from the participants.

Western ethnic-minority villagers have relatively low levels of educational attainment; among those who speak the local ethnic language as their first language, data collection is more difficult. During the data collection process, there may be potential biases, especially social desirability bias. To reduce this bias, we implemented the following measures: First, during the field survey, we ensured anonymity of questionnaires to encourage truthful responses. Second, we designed neutral and non-leading questions to minimize adjustments due to social expectations. Third, we trained surveyors to maintain neutrality and avoid influencing responses through tone or non-verbal cues. Additionally, given our focus on ethnic-minority villagers in western China, we addressed specific potential biases inherent in the questionnaire process. For example, younger, more skilled, younger villagers may have been more inclined to participate, while older villagers, those facing communication barriers, or those in geographically remote villages might have been underrepresented. To address these issues, we drew on field research methods and adopted multiple strategies during the sample collection process: First, before implementing the survey, we stayed in the village for 3–5 days, engaging with local villagers daily, such as greeting them, chatting, and participating in their activities. Once we established a good rapport with the villagers and became familiar with their language habits, we began the questionnaire survey. During the survey, if necessary, we would explain the meaning of the items using vocabulary that the villagers could easily understand. Second, we consulted community leaders to understand the villagers’ participation in PPT, ensuring that the survey covered villagers of different age groups and educational backgrounds. Third, we employed weighted data analysis techniques to minimize the impact of sample bias on the research results. In addition, we also considered time bias, cultural bias, and selection bias. To address these biases, we conducted a questionnaire pre-test and back-translation, collected data at multiple time points, and incorporated culturally sensitive design while expanding the sample size. These measures helped improve the accuracy and reliability of the research findings.

Previous methods for studying MOA include multiplicative models that focus on the complementary relationship between the three variables of MOA [43,53], fsQCA methods for complex causal relationships between the three variables and specific results [84], regression analysis of the interaction relationship between variables [50], Structural Equation Modeling (SEM), etc. However, based on the purpose and needs of the study, SEM has been used to test the correlations among factors. This comprehensive multivariate statistical analysis method allows for the simultaneous examination of all relationships among variables in a conceptual model [85]. Prior studies have shown that SEM samples must be at least 100 or above to be reliable [86,87]. Based on the proposed method of data analysis, 248 questionnaires were distributed; when invalid questionnaires were excluded, 201 valid questionnaires were obtained, with an efficiency rate of 81.05%.

Most participants (64.68%) were 30–49 years old; 78.12% were educated below high school level. In relation to tourism occupations, most worked at B&Bs or rural hotels or earned a living from catering, snacks, or handicraft sales. They had been engaged in the tourism business for less than 5 years. In relation to tourism knowledge, the villagers were more likely to learn about attracting customers, promotions and sales, online sales, and customer service. Most learned by visiting and studying, searching the Internet, questioning others, chatting face-to-face, and being trained by experts. The survey included residents of different genders, ages, education levels, ethnicities, and occupations; the sample was highly random and representative, ensuring the reliability of the study.

### Measures

This study has measured all items using a 5-point Likert scale. The scales used to measure items in the research model were mainly adopted from the literature. A total of 19 measurement questions were included.

#### Motivation.

In ethnic-minority villages, individual motivation is driven not only by personal goals and interests but also by community values, social relationships, and cultural traditions [88]. Ethnic-minority cultures emphasize collective interests and mutual support, with individual behavior often aligning with the community’s overall goals [89]. Therefore, when selecting scales to measure motivation, we specifically focused on whether they could reflect an individual’s intrinsic motivation within the community context. In conclusion, the scales used to measure motivation were drawn from McLure and Faraj (2005) [90], Davenport (1998) [91], and Siemsen (2008) [53]. Items included: “earning from others will help me acquire knowledge about tourism services;” “I can earn more money and improve my living conditions by engaging in tourism business.” The Cronbach’s α for this part of the study was 0.62.

#### Opportunity.

The scales used to measure knowledge transfer opportunities were drawn from Venkatesh and Davis (2000) [92] and Cao (2012) [93]. Items included: “I can learn about tourism services from many sources and in different ways;” “Experts, scholars and students will teach us knowledge and skills about tourism services.” The Cronbach’s α for this part of the study was 0.71.

#### Ability.

Knowledge absorptive capability refers to the ability of villagers to identify and learn relevant knowledge about the production and services of rural tourism products, transform it into personal knowledge, and apply it in rural tourism practices. The scales used to measure ability were drawn from Szulanski (1996) [94], Siemsen (2008) [53], and Chang et al. (2012) [95]. Items included “I can apply the external knowledge I’ve learned to actual travel business operations.” The Cronbach’s α for this part of the study was 0.63.

#### Knowledge Transfer Effect.

The scales used to measure the knowledge transfer effect were drawn from Kostova (1999) [96] and Zhou and Zhou (2018) [24]. Items included, “Through learning in various ways, I have learned a lot of knowledge that is useful for tourism practice,”; “Through learning, I am satisfied with the effectiveness of learning knowledge for tourism practice” and four other questions. The Cronbach’s α for this part of the study was 0.82.

#### Trust.

Given the cultural uniqueness of ethnic minorities, trust-building is often closely linked to community relationships, family ties, and traditional customs [97]. Ethnic-minority cultures emphasize collectivism and mutual assistance, tending to be more friendly and trusting toward outsiders, such as tourists [98]. This cultural context led us to prioritize trust measurement scales reflecting both mutual support within the community and trust toward outsiders. Therefore, the scales used to measure trust were drawn from Hsu et al. (2007) [99] and Dallago et al. (2009) [100]. Items included: “I trust government employees and public welfare organizations to give me valuable advice on tourism operations;” “I trust experts, scholars, and students who are willing to help me;” and “I trust them to help me with my work.” “I trust experts, scholars, and students who will help me improve my business performance.” The Cronbach’s α for this part of the study was 0.79.

## Result

### Goodness-pf-fit test

Before testing the model hypothesis, we explored the influence of three factors—motivation, opportunity, and ability—on the effect of tourism knowledge transfer through the maximum likelihood estimation method. A goodness-of-fit test was carried out on the hypothetical model. The fit indicators used to test the goodness-of-fit of the sample data and conceptual model included the absolute fit indicator, value added fit indicator, and parsimonious fit indicator (see Table 1). According to the goodness-of-fit test, the X2/df is 2.147, the GFI is 0.913, the AGFI is 0.872, the RMSEA is 0.076, the NFI is 0.849, the TLI is 0.888, the CFI is 0.911, the IFI is 0.913, the PNFI is 0.674, and the PGFI is 0.622, where the values of GFI, CFI, and IFI are greater than 0.9, the value of RMSEA is less than 0.08, and the value of X2/df is less than 3. The values of GFI, CFI, and IFI are greater than 0.9; the value of RMSEA is less than 0.08; and the value of X2/df is less than 3. Since most indicators are well fitted, the structural equation model fit constructed in this paper is considered to be of an acceptable level in relation to related studies [101]. Most indicators met the fit criteria, indicating that the structural equation model was generally valid.

**Table 1:** Hypothesis model goodness-of-fit tests.

Fitted Indicators	Absolute Fit Indicators				Value Added Fit Indicators				Simple Fit Indicators	
X^2^/df	GFI	AGFI	RMSEA	NFI	TLI	CFI	IFI	PNFI	PGFI
Fit criteria	(1,3)	>0.90	>0.90	<0.08	>0.90	>0.90	>0.90	>0.90	>0.50	>0.50
Initial model	2.147	0.913	0.872	0.076	0.849	0.888	0.911	0.913	0.674	0.622

### Descriptive statistics

The means, standard deviations, correlations and scale reliabilities between the variables are shown in Table 2. This table shows a positive correlation between knowledge transfer opportunity and transfer motivation (r = 0.52, p < 0.01), between knowledge transfer opportunity and knowledge receiving capacity (r = 0.55, p < 0.01), and between knowledge transfer opportunity and knowledge transfer effectiveness (r = 0.58, p < 0.01). These results provide initial support for the study hypotheses.

**Table 2:** Means, standard deviations and correlations.

Variables	Mean	SD	1	2	3	4	5	6	7	8
1. Gender	1.51	0.50								
2. Age	3.48	1.12	-0.06							
3. Educational level	2.75	1.07	-0.22^**^	-0.28^**^						
4. Motivation	3.82	0.57	-0.17^*^	0.06	0.07	(0.62)				
5. Opportunity	3.70	0.72	-0.13	0.12	0.15^*^	0.52^**^	(0.71)			
6. Ability	3.65	0.60	-0.15^*^	0.04	0.16^*^	0.28^**^	0.55^**^	(0.63)		
7. Knowledge Transfer Effect	3.95	0.59	-0.15^*^	0.08	0.08	0.45^**^	0.58^**^	0.45^**^	(0.82)	
8. Trust	3.82	0.65	-0.13	0.09	0.02	0.53^**^	0.58^**^	0.49^**^	0.57^**^	(0.79)

**Notes:** N = 201. M = mean, SD = standard deviation.

** p < 0.01,

* p < 0.05. Cronbach’s a in italics. Gender: 1 = male, 2 = female. Education: 1 = primary school education or below, 2 = junior high-school educational level, 3 = high-school educational level, 4 = junior college degree, 5 = bachelor’s degree and above

### Reliability and validity testing

Using SPSS 26.0, we analyzed the reliability and validity of the questionnaire data. The analysis results are shown in Table 3:

**Table 3:** Reliability and validity testing results.

Dimension	Cronbach’s α	KMO Value	Bartlett’s Test of Sphericity		
Chi-square Test	Degrees of Freedom	Significance
Motivation	0.615	0.632	71.558	3	0.000
Opportunity	0.712	0.641	127.967	3	0.000
Ability	0.625	0.647	71.797	3	0.000
Knowledge Transfer Effect	0.821	0.803	275.878	6	0.000
Trust	0.791	0.642	215.551	3	0.000
Overall Scale	0.890	0.851	1299.865	120	0.000

The Cronbach’s α coefficient for the overall scale reached 0.890, and the Cronbach’s α coefficients for individual scales ranged from 0.615 to 0.821, all greater than 0.6, indicating high reliability of the scales. The validity test for the overall scale showed a KMO value of 0.851, with KMO values for individual scales ranging from 0.641 to 0.803, and Bartlett’s test of sphericity for all scales was significant at the 1% level, indicating high validity of the scales.

The Cronbach’s alpha values for motivation (α = 0.62) and ability (α = 0.63) are relatively low. Item Analysis and Confirmatory Factor Analysis (CFA) are conducted to ensure the reliability and validity of these constructs. Item Analysis results show that the corrected item-total correlations for all items were above the suggested threshold of 0.30, with most exceeding 0.5, indicating strong correlations between each item and the total score. Therefore, no items are removed. Confirmatory Factor Analysis (CFA) is used to assess construct validity and examine item cross-loadings. The results show that most variables had factor loadings greater than 0.4 or 0.5 on one factor, with lower loadings on other factors. This suggests a clear relationship between the variables and factors, with no significant cross-loadings. The model fit indices were also within an acceptable range (CFI = 0.915, TLI = 0.875, RMSEA = 0.074), supporting the construct validity of the measurement model.

### Hypothesis testing

The conceptual model fitted well. On this basis, the research hypotheses were verified through a parameter estimation and path analysis between the endogenous and exogenous variables of the model, as shown in Table 4. The results show that the standardized path coefficient of the knowledge transfer opportunity and knowledge transfer effect was 0.72, with the p-value significant at the 0.001 level. Thus, hypothesis H2 is valid: knowledge transfer opportunity directly and significantly affected the knowledge transfer effect. The two standardized path coefficients—from knowledge transfer motivation to knowledge transfer opportunity and from knowledge reception ability to knowledge transfer opportunity—were 0.636 and 0.689 respectively, with p-values significant at the 0.001 level. Therefore, hypotheses H1 and H3 are valid, indicating that knowledge transfer motivation and knowledge absorptive capacity directly and significantly affect knowledge transfer opportunity, and indirectly affect knowledge transfer effect through knowledge transfer opportunity, with knowledge absorptive capacity having a greater impact on knowledge transfer opportunities.

**Table 4:** Table of structural model path coefficients.

	Unstandardized				Standardized
	Estimate	S.E.	C.R.	P	Estimate
Opportunity ← Motivation	0.643	0.115	5.576	^***^	0.644
Opportunity ← Ability	0.562	0.095	5.935	^***^	0.702
Knowledge Transfer Effect ← Opportunity	0.718	0.104	6.871	^***^	0.720
M1 ← Motivation	0.782	0.144	5.423	^***^	0.516
M2 ← Motivation	0.722	0.122	5.924	^***^	0.593
M3 ← Motivation	1				0.691
O1 ← Opportunity	0.943	0.134	7.027	^***^	0.586
O2 ← Opportunity	1.160	0.153	7.584	^***^	0.641
O3 ← Opportunity	1				0.688
TE1 ← Transfer Effect	1				0.738
TE2 ← Transfer Effect	0.898	0.096	9.384	^***^	0.743
TE3 ← Transfer Effect	1.003	0.108	9.275	^***^	0.733
TE4 ← Transfer Effect	0.887	0.104	8.538	^***^	0.667
A1 ← Ability	0.511	0.088	5.812	^***^	0.531
A2 ← Ability	0.623	0.108	5.795	^***^	0.529
A3 ← Ability	1				0.737

**Note:**

*** P < 0.001

The moderating effect determines “the effect of a variable on the relationship between two other variables”. In this study, a product indicator was used to test the moderating effect of trust on the relationship between knowledge transfer opportunities and knowledge transfer effects by constructing an interaction multiplier. The standardized path coefficient of the interaction multiplier “transfer opportunity*trust” and the knowledge transfer effect was found to be 0.114, with a p-value of 0.145. Therefore, H4 did not hold, indicating that the trust factor did not have a significant positive moderating effect between knowledge transfer opportunity and knowledge transfer effect among ethnic-minority villagers in western China.

## Discussion

### Theoretical implications

This study examines the factors that influence the transfer of tourism knowledge among villagers in western China’s ethnic-minority regions, based on the MOA framework. Using a structural equation modeling approach, it empirically verifies the effects of motivation, opportunity, ability, and trust on the knowledge transfer effect and draws the following conclusions:

#### First, opportunity acts as the main driver in the transfer of tourism knowledge to villagers in western China.

This study confirms that opportunity acts as the main driver in the MOA model in specific contexts. While motivation is generally considered the direct driver of action in the MOA framework, opportunity is the limiting environmental factor that influences motivation and behavioral activity; ability is the level of skill required to drive behavior. The three interact to induce individual behavior [102]. However, some researchers have questioned the prominence of motivation through empirical studies. If we recall [53] assertion that a constraint variable model based on the MOA framework has been constructed, confirming that constraints among MOA variables ultimately determine the occurrence of behavior and that motivation is not always the primary constraint in the MOA framework, then the above results leave us in no doubt that opportunity becomes a constraint in the MOA framework during the transfer of tourism knowledge to ethnic-minority villagers in western China. As the villagers transform their lives from traditional farming patterns to tourism service work to escape poverty, they must invest time, energy, and material, bearing financial costs and taking huge investment risks. Given these risks, it is essential for them to learn about tourism practices to adapt to the new lifestyle. This also echoes the arguments of Rosalina et al. (2021) [12] that although rural residents have a strong incentive to transfer knowledge, most are isolated; they live at high altitudes and with less access to transportation, lower levels of education, and fewer opportunities to learn and experience tourism than people living in urban areas. This makes direct contact between villagers and knowledge sources invaluable, causing knowledge transfer opportunities to become the main driver of knowledge transfer effects. Among various knowledge transfer opportunities, the most influential factors are external forces, such as experts, students, journalists, tourists, and government staff. The least influential factor is the richness of knowledge transfer channels, indicating that most villagers rely mainly on direct contact with external knowledge sources to acquire tourism knowledge skills; their access to knowledge transfer is still relatively homogenous. In China, the central government plays a steering role in guiding rural tourism in the desired direction, while local governments play a serving role by managing tourism practices and coordinating with businesses and residents to provide services and solve problems [64]. Local governments in western China should create more opportunities for guest–host knowledge transfer in the practice of rural tourism management.

#### Second, knowledge transfer motivation has a positive and significant effect on knowledge transfer opportunities.

Rural tourism is hindered by the potential hosts’ lack of interest in or awareness of tourism [12]. Self-determination theory argues that human behaviors may be encouraged by externally induced and internally evoked incentives [103], while autonomous motivations are more likely to result in more positive outcomes [104]. The knowledge transfer motivation of villagers in ethnic-minority regions in western China includes both autonomous motivations (a strong desire to improve livelihood patterns) and external motivations (knowledge sources that interact in profound ways with local villagers’ occupational transitions). Luo et al. (2022) [105] claimed that cognition directly impacts rural people’s willingness to be involved in rural tourism services. When villagers are faced with modern civilization, poverty poses unexpected difficulties and affluence often means an increase in dignity, as well as an improved quality of life. Local community culture guides the thinking and behavior of villagers and makes a crucial contribution to rural-tourism destination competitiveness [106]. To expand their horizons and acquire tourism knowledge, villagers use internal motivation to change careers and build a learning community culture.

#### Third, knowledge absorptive capacity has a positive and significant effect on knowledge transfer opportunities.

The enhanced knowledge absorptive capacity of villagers significantly contributes to their ability to comprehend environmental changes, process information, and identify, acquire, and apply knowledge, enhancing their skills and expertise in tourism services. Knowledge absorptive capacity impacts knowledge transfer opportunities more than motivation because it enables villagers to seize opportunities [107]. Knowledge absorptive capacity is the ability to identify and acquire external knowledge. Of particular note is that among knowledge absorptive capacity evaluation factors, the most influential is professional knowledge sources perception ability, i.e., the ability to find experts who can solve problems quickly and satisfactorily. In practical terms, villagers maintain that challenges encountered in tourism operations become less daunting once they can access experts who provide the necessary knowledge and solutions. Moreover, knowledge absorptive capacity and prior knowledge are equally influential [108]. In various forms, prior knowledge positively impacts knowledge absorptive capacity, encouraging individuals to internalize external knowledge.

#### Fourth, trust only slightly enhances the transfer of tourism knowledge to villagers.

Most academics believe that trust plays an important role in knowledge transfer by increasing participants’ willingness to engage and reducing difficulties [109]; high levels of trust strengthen communication channels, increase communication frequency, and deepen knowledge exchange, thus building transferable knowledge and making the transfer process easier [110]. The present study shows that trust does not positively regulate the relationship between knowledge transfer opportunities and knowledge transfer effects. The present finding diverges from prior research that typically conceptualizes trust as a moderating factor [81,82]. This divergence may be attributed to the specific research context and sample composition. Ethnic-minority communities practice and respect mutual help, honesty, and courtesy. Influenced by traditional culture of respecting teachers and valuing education, they respect external knowledge sources (e.g., scientists and professors) [111]. Minority villagers have a high level of trust in external knowledge sources, so continued increases in trust did not motivate villagers to improve knowledge transfer effects. As trust does not moderate the positive influence of knowledge transfer opportunities on knowledge transfer effects, continuously increasing trust does not produce more positive feedback. This aligns with prior research indicating that high trust levels may diminish the discernible moderating effect of trust within the knowledge transfer dynamic [112]. Furthermore, the surveyed areas in this study have experienced tourism development for over a decade, and in some cases, nearly three decades. Local villagers frequently interact with external stakeholders, fostering long-standing communication and cooperative relationships with experts, poverty alleviation organizations, and tourists. This established familiarity contributes to a high degree of trust in these knowledge sources. Consequently, the moderating role of trust in knowledge transfer within ethnic-minority villages during the initial phases of tourism development warrants further empirical examination.

### Practical implications

Based on the above analysis, this study makes the following recommendations.

#### First, villagers should develop professional skills by acquiring new tourism knowledge.

The pattern of rural tourism in western Chinese ethnic communities has gradually changed from poverty-alleviation to leisure and holiday tourism [113]; tourism services have also changed their business philosophies and operations. Innovation carriers recommend non-formal education as a comprehensive strategy that can meet the needs of rural communities, providing lifelong learning skills that can be applied to tourism [114]. Adult learning is strongly linked to and integral to survival. Adult learning is task-oriented and hands-on participatory. Villagers involved in rural tourism should understand that knowledge transfer is an evolving and cyclical process, requiring adaption to new professional roles, a forward-looking approach and a sense of crisis. They should take advantage of non-formal educational opportunities to acquire knowledge and skills and respond to increasingly fierce market competition by adding value through customer orientation, quality management, product diversification and marketing [115]. To respect the cultural and linguistic diversity of ethnic minorities, non-formal education can be designed with multilingual and multicultural content. A bilingual teaching approach, integrating local culture and traditional knowledge, can align educational content more closely with villagers’ actual needs [14]. It is encouraged that ethnic-minority tourism experts familiar with local culture and language explain tourism knowledge and skills to villagers. For example, Professor Wu, a local Miao tourism expert, conducted rural tourism planning for the Longji Rice Terraces Scenic Area, teaching local villagers tourism service knowledge and skills. Ethnic minority experts can teach in a way familiar to villagers based on local circumstances, reducing barriers caused by language and cultural differences. Moreover, in the Binglang Valley Li and Miao Cultural Village in Baoting Li and Miao Autonomous County, local communities organize experts to teach villagers how to provide new cultural experience tourism products, such as traditional Li ethnic song and dance performances and handicraft demonstrations. Villagers acquire tourism service knowledge and skills through “learning by doing”. The learning content reflects respect for and inheritance of Li ethnic culture, and villagers find it easy to learn and are willing to accept it.

#### Second, the government should motivate villagers to transform their occupations.

In China’s minority areas, PPT is a “top-down” approach [116]. Restricted by the rural location, education level, and interpersonal relationships, rural residents need to be more information-insensitive. Lagging access to information and poor information channels are stumbling blocks that prevent them from understanding external changes and keeping pace with the times. The number of rural residents participating in rural tourism is limited, and the enthusiasm for participation is not high because of the lack of information, communication channels, and financial resources for villagers. The government should establish channels for villagers to communicate smoothly with the outside world. The government should continue to improve rural infrastructure, including the Internet. Villagers also need easy access to free information via the Internet [117]. The government should also support tourism business activities through financial and taxation policies and infrastructure construction [118]. It should publicize examples tourism enrichment and motivate villagers to learn by doing, accumulate knowledge through field experience and embrace occupational transformation.

#### Third, government and non-profit pro-poor organizations should strengthen the training and learning of villagers.

Since education levels can influence tourism participation [119], government and non-profit pro-poor organizations should focus on improving the villagers’ ability to participate and strengthening the training and learning of villagers, such as catering skills, service skills, communication skills, and competition skills, to improve the level of tourism services and competitiveness. They should build contacts between local people and external knowledge sources, encouraging experts, journalists, and tourists to impart practical and advanced tourism knowledge. Moreover, recognizing the inherent diversity among villagers, including differences in experiences, interests, cognitive styles, and abilities, is essential. It underscores the need to tailor educational endeavors’ content, methods, approaches, and evaluation criteria to cater to individual preferences and learning profiles. The government, educational institutions, and social organizations must be committed to providing villagers with well-suited learning opportunities while ensuring equal access for comprehensive participation.

#### Fourth, the government should support the establishment of community knowledge-sharing networks.

To strengthen coordination and collaboration among stakeholders and create more effective knowledge-sharing networks, the government should support the establishment of collaboration platforms and regular communication mechanisms. For example, a collaborative committee composed of representatives from the government, non-governmental organizations, and businesses could be formed to develop and supervise tourism development plans, ensuring effective collaboration on knowledge sharing and tourism development. The government should encourage the creation of learning communities promoting knowledge sharing among small rural tourism enterprises and emphasize the role of first-generation rural tourism entrepreneurs and other tourism elites, encouraging them to share advanced tourism knowledge, experiences, or lessons [30]. Additionally, the government can incentivize businesses and NGOs to establish knowledge-sharing partnerships with villagers through policies such as tax breaks and financial subsidies, while promoting cross-village collaboration projects that facilitate experience exchange and resource sharing among different villages [120]. In cross-village collaboration projects, businesses or NGOs, with their expertise and technical support, can help villagers improve tourism service skills and management abilities, thereby promoting resource complementarity and collaborative development, achieving “big villages supporting small villages, strong villages supporting weak villages”.

#### Fifth, the government should help villagers learn tourism service knowledge online.

Online learning can effectively overcome geographic and infrastructure barriers, promoting knowledge transfer [121]. As rural internet infrastructure improves and mobile communication devices (such as smartphones) become more widespread, the government should encourage and assist villagers in learning tourism service knowledge through various online channels [122]. Specific measures include: 1) Utilizing self-media platforms. Encouraging villagers to use platforms like TikTok, Kuaishou, and Little Red Book to acquire and share tourism service knowledge. These platforms offer rich tourism information and practical experiences, enabling interactions with tourists, tourism businesses, and experts. 2) Participating in online communities: Guiding villagers to join online communities, such as BBS or blogs, to exchange experiences and gain practical tourism service knowledge with other practitioners and experts. 3) Using mobile learning platforms: Promoting mobile learning platforms like Tourism Micro-Lecture, China Tourism Education Online Training Platform, and China Tourism Online Education, which offer professional tourism service training to help improve villagers’ service skills.

#### Sixth, knowledge transfer impacts economic empowerment, social mobility, the preservation of cultural heritage, and community cohesion.

Knowledge transfer not only improves villagers’ tourism service levels but also positively impacts economic empowerment, social mobility, preservation of cultural heritage, and community cohesion in ethnic-minority villages [14]. This is reflected in: 1) Knowledge transfer enables villagers to acquire more advanced tourism management knowledge and skills, improving tourism service quality and operational efficiency, thus significantly boosting tourism income. For example, in the Longji Rice Terraces scenic area, villagers’ per capita tourism income increased from 33% of total income in 2002 to 92% in 2019 due to knowledge transfer. 2) Knowledge transfer allows villagers to gain more economic benefits and social recognition from tourism management. The development of rural tourism provides villagers with opportunities to interact with the outside world, expanding their economic, social, and cultural networks, enhancing social mobility. 3) Knowledge transfer has promoted the establishment of mechanisms for protecting rural natural resources and cultural heritage [123]. Through interactions with tourists and experts, villagers not only enhance the quality of tourism products and services but also learn about tourists’ strong interest in natural landscapes and ethnic-minority cultures. Through communication and cooperation with governments, tourism companies, and experts, villagers have organized and implemented resource conservation actions. For example, in the Longji Rice Terraces Scenic Area, a community protection mechanism was gradually developed through guest-host knowledge transfer, with all parties jointly maintaining the terraced landscape and cultural heritage. 4) Guests-hosts knowledge transfer not only facilitates the acquisition of new tourism knowledge by villagers but also promotes knowledge exchange and business cooperation among villagers, strengthening community cohesion and promoting sustainable community development.

#### Seventh, knowledge transfer models need to be continuously adjusted and optimized.

With the development of the tourism industry, knowledge transfer models require continuous adjustment and optimization [39]. As rural tourism in western ethnic regions matures, the knowledge transfer model will shift from guest-host knowledge transfer to community sharing, from traditional training to digital learning, and from knowledge input to knowledge innovation. By establishing learning communities and knowledge-sharing platforms, villagers can more widely exchange and share tourism management experiences, raising the tourism service standards across the community. As villagers’ digital literacy improves, knowledge transfer will increasingly rely on digital platforms. For example, through online courses, mobile learning platforms, and social media, villagers can more easily access tourism management knowledge and skills. As the western ethnic-minority rural tourism industry develops, villagers will not only absorb external knowledge but also gradually combine it with local realities, creating new models and methods suitable for local tourism development. Furthermore, governments and social organizations should continuously attend to the needs and interests of villagers to ensure the effectiveness and sustainability of knowledge transfer models [14].

#### Eighth, implications of this study for other remote or marginalized regions globally.

This study applies the MOA framework to ethnic-minority regions in western China, revealing the unique impact of motivation, opportunity, and ability on knowledge transfer and tourism development. These findings not only offer new research perspectives for western China but also provide valuable references for other underdeveloped or culturally distinct regions facing similar challenges, such as parts of Africa, Southeast Asia, and Latin America. Specifically, in many remote areas of Africa, poverty, lack of educational resources, and cultural diversity make knowledge transfer more challenging [124]. How to improve the opportunities, motivation and ability of minority villagers to transfer knowledge becomes particularly important. Edwards et al. (2019) utilized an evidence mapping methodology to develop a framework of knowledge transfer strategies, outcomes, facilitators, and barriers based on social and health sciences literature. Their findings indicated that effective knowledge transfer in African countries is significantly hindered by challenges such as poverty, limited resources, and insufficient government awareness of the importance of knowledge transfer [125]. In rural Southeast Asia, the development of tourism is often limited by inadequate infrastructure and cultural differences [126]. The MOA framework employed in this study provides valuable insights for effective knowledge transfer strategies in remote or marginalized regions, while also highlighting the critical role of trust in cross-cultural communication. Many remote areas in Latin America also face issues such as underdeveloped education and infrastructure [127]. The findings of this study provide practical guidance to these regions, particularly in how governments and non-profit organizations to enhance knowledge transfer method, through strategies like establishing community knowledge-sharing networks and fostering online learning and knowledge innovation, and prioritizing the needs and interests of the poor populations to ensure knowledge transfer effectiveness.

## Limitations and future research

The study has limitations that future studies should address. First, to improve the generalizability of findings, future studies should expand the sample and region beyond ethnic-minority villages in western China. Research could broaden the scope to include villagers engaged in tourism within ethnic-minority areas from more regions across China or conduct cross-national comparative studies. A broader geographical sample could better capture commonalities and differences in factors affecting knowledge transfer, such as trust, motivation, opportunity, and ability, among villagers from ethnic-minority villages in different countries. This would help identify global best practices and general trends, providing more comprehensive references for policy-making and PPT practices. Second, future research should consider using multi-source data collection methods, such as collecting primary data through observation and interviews, and secondary data through reviewing literature, analyzing statistical data, and consulting expert opinions, to improve the comprehensiveness, accuracy, and reliability of the research findings. In terms of research methods, qualitative approaches should be employed, such as longitudinal single-case studies, multiple-case studies, grounded theory, focus groups, participatory evaluation, and ethnographic research, or a combination of qualitative and quantitative methods, to reveal the complexity and dynamics of PPT knowledge transfer and analyze in-depth the long-term impact of knowledge transfer on community development. Third, given the long-term nature of poverty alleviation and sustainable enrichment through tourism, researchers must continue to explore ways to innovate knowledge transfer to adapt to evolving market demands and technological advancements. Digital technologies offer opportunities to bridge the knowledge gap in remote areas; researchers should explore the potential of digital tools (e.g., online training platforms, mobile applications, online collaboration platforms, AI, VR, AR, big data, and information-sharing platforms) to provide forward-looking suggestions for policy-making and practices. Fourth, given that western China’s ethnic regions serve as a migration and exchange corridor marked by multiethnic cultures and multireligious beliefs, future research should explore how ethnic cultures and religious beliefs influence local people’s external perceptions, emotions, values, ethics, and spiritual identity as they adopt advanced knowledge systems imported from the outside world. Fifth, future studies should deeply explore the impact of gender dynamics (e.g., gender roles, educational opportunities, social participation and mobility, gender roles in family and marriage) on knowledge transfer in ethnic-minority communities from an intersectional perspective. This would not only promote the equity and effectiveness of knowledge transfer but also help foster the sustainable development of the social and economic structures of ethnic minority villages.

## Supporting information

S1 Dataset MOA Questionnaire Data. We have shared the collected data; for details, please refer to: https://doi.org/10.6084/m9.figshare.27078997. (XLS)
